# The Differential Diagnosis of Præfrontal and Cerebellar Tumours

**Published:** 1901-02-16

**Authors:** Arthur Maude


### Hospital Clinics and Medical Progress.

### THE DIFFERENTIAL DIAGNOSIS OF PRÆFRONTAL AND CEREBELLAR TUMOURS.

By Arthur Maude.

Nellie I., aged 21, has been under observation since May 21st, 1900. She had then been complaining for some weeks of failing sight and severe headache, especially in morning. She was seen during my absence by Dr. Ironside Moir, who noted distinct retinitis about both yellow spots, blurred discs with commencing atrophy, no great loss of vision ($V\frac{6}{12}\,\bar{c} + \cdot 5D\,\frac{6}{5}$). Nystagmiform movements on extreme lateral deviation of eyeballs, and on upward movement. Asthenopia marked: V J1 at 12 inches with difficulty, readily with + 1 (right and left). Latent concomitant strabismus. Cardiac hypertrophy; pulmonary hæmic murmur; no albuminuria.

In August, 1900, first seen by me. Condition of vision and discs unchanged. Attacks of recurrent vomiting had begun; very high tension pulse. Heart showed marked hypertrophy, with excited action. Loud double murmur over pulmonary area, passing into a soft hæmic murmur upwards; harsh aortic double murmur, especially marked with systole.

*November 5th.*—First severe attack of vertigo, sickness, and unconsciousness. Position of headache is suprafrontal, equal on both sides, and occipital just above occipital protuberance, bilateral also. Patellar reflex is quite absent both sides. She remained unconscious about a week. On recovery lapsed into a condition of pleased, chuckling inertia; very slow cerebration. No squint, not much headache, no paresis except of lower limbs. Gait very feeble, doubtful tendency to fall towards left side. She had another attack of partial unconsciousness.

*January 21st.*—Drooping of right eyelid with slow, upward movement of lid first noticed. This is the first definite paresis of any group of muscles. There is nothing remarkable in the family history. No signs, history, or suspicion of syphilis. The girl is very stunted, almost dwarfish, in growth, and all the family are small, ill-developed, and under-sized. She is not markedly anæmic, has no renal disease, and shows no signs of lead poisoning. There has been no disease of the middle ear, or any condition likely to lead to chronic cerebral abscess.

Here we have a case which by the end of August, 1900, could be unhesitatingly pronounced one of intracranial tumour. For months the only tangible symptoms were an intense double optic neuritis and recurrent headache which might have been due to intrinsic optic neuritis, coupled with a low degree of hypermetropia. In August the recurrent attacks of sickness gave no room for doubt, but even now the question of the position of the tumour demands careful summing up of the evidence at our command. There are as yet no local symptoms, neither palsy nor spasm; we have therefore to review the evidence presented in the light of recent knowledge. Two important "symposia" on the subject have been held in England recently: one a discussion at the annual meeting of the British Medical Association in 1898, led by Dr. Ferrier; the other a discussion at the Neurological Society, opened by Dr. Charles Beevor. To collate the opinions and impressions given at both these meetings is absolutely necessary for any thoughtful estimate of such a difficult and obscure case.

Any case of undoubted intracranial tumour which has presented general signs for some time without paralysis is sure to fall under the headings of "cerebellar" or "præfrontal." It is in the differential diagnosis of frontal and cerebellar tumours that the greatest difficulty occurs. The excellent resemblance afforded clinically by tumours in such widely separated portions of the brain is explained by the anatomical connection of the parts. Each præfrontal lobe is connected with the lateral lobe of the cerebellum on the opposite side by fibres passing downwards in the anterior part of the internal capsule, on the inner side of the crus cerebri, and in the superior cerebellar peduncle.

To take the leading symptoms in our case seriatim we begin with the earliest, "optic neuritis." This may well be described as of an early intense form. Gunn, in an admirable note on the point ("Brain," 1898, p. 334), enforces the fact, now well known to experts, that cerebellar tumours are prone to excite a peculiarly intense neuritis, with great engorgement of the papillæ, and marked œdema of the surrounding retina, with not uncommonly a macular stellate figure, similar to that seen in albuminuric retinitis. In cerebellar tumour the neuritis is usually bilateral, with little difference in degree between the two sides; in præfrontal tumour, on the other hand, there is a marked tendency to affection of one disc (Williamson, "Brain," 1896, p. 363). The neuritis in our patient is only slightly more marked on the right side. Our patient is hypermetropic, but her hypermetropia is symmetrical. The question has been raised whether hypermetropes are not peculiarly liable to optic neuritis, and that in cases of asymmetrical hypermetropia the more hypermetropic eye has a more developed neuritis. The evidence which has been discussed by Gowers and Gunn is highly suggestive, but must be considered as yet subject to correction.

It is not as much the locality of intracranial tumours which influences the production of optic neuritis as the vagaries of their growth. Rapid growth and sudden congestion, or venous stasis, a great increase of cerebro-spinal fluid in subcerebral spaces, or the production of basal meningitis are the factors which lead to papillitis. It is the position of the cerebellum to the venous outlets of the brain, and the large meningeal spaces at the base, which render cerebellar tumours so productive of optic neuritis.

The position of the headache in our patient is negative: it is suprafrontal, beginning usually on the right side, and also is felt at the occiput on both sides. Frontal and occipital headache often coincide both in cerebellar and præfrontal tumour (Williamson). However defined the pain may be in the occipital region, when tumour of the cerebellum is present it is exceptional to miss headache of the frontal region as well; if limited to our frontal area it selects the opposite to the side of the tumour.

Tenderness of the skull-cap is a less constant sign than localised headache. It is generally supposed to exist in cases of cortical tumour, and from the position of the cerebellum and the thickness of the occiput is comparatively rare in cerebellar tumour. Tenderness of the skull is not present in our case. If present in the frontal region it is a valuable factor in aiding localisation. There is no loss of smell in our patient. This is, of course, negative evidence. Anosmia is rare in all cases of cerebral tumour, but when present is strongly in favour of a præfrontal site (Williamson). It is noted in seven out of seventeen cases of præfrontal tumour ("Brain," 1896, p. 363). But we must remark that bilateral loss of smell has been described in an extra medullary cerebellar growth (Beevor, *loc. cit.*, p. 295). The complete absence of motor symptoms, where general symptoms of intracranial tumour are obvious, is strong evidence in favour of the præfrontal position of the lesion: it is in the "silent area." If, later, paralysis ensues, it may be due to extension backwards from the præfrontal to the motor area of the cortex, or the groups of motor fibres passing down from the cortex towards the internal capsule; their extension may not be a direct invasion by a defined growth, but may be infiltration by a diffuse growth, or softening of otherwise healthy brain substance surrounding a defined growth.

*(To be continued)*

